# Travis County Medical Society

**Published:** 1904-01

**Authors:** 


Travis County Medical Society.—Annual election of officers January 9th, inst. Dr. Ralph Steiner was elected president; Dr. H. B. Hill, vice president; Dr. M. M. Smith, secretary; Dr. W. A. Harper, secretary.

